# Association of Microbial Networks with the Coastal Seafloor Macrofauna Ecological State

**DOI:** 10.1021/acs.est.4c12464

**Published:** 2025-04-11

**Authors:** Tonje Nilsen, Ragnhild Pettersen, Nigel Brian Keeley, Jessica Louise Ray, Sanna Majaneva, Morten Stokkan, Anja Hervik, Inga Leena Angell, Lars Gustav Snipen, Maud Ødegaard Sundt, Knut Rudi

**Affiliations:** † Norwegian University of Life Sciences, Ås 1433, Norway; ‡ Akvaplan-niva, Tromsø 9007, Norway; § Institute of Marine Research, Tromsø 9296, Norway; ∥ Aqua Kompetanse AS, Flatanger 7770, Norway; ⊥ STIM AS, Lofoten 8370, Norway

**Keywords:** 16S metabarcoding, shotgun metagenomics, seafloor sediment, environmental monitoring, aquaculture, macrofauna, sulfur oxidizers, ammonium oxidizers, Norway, Iceland

## Abstract

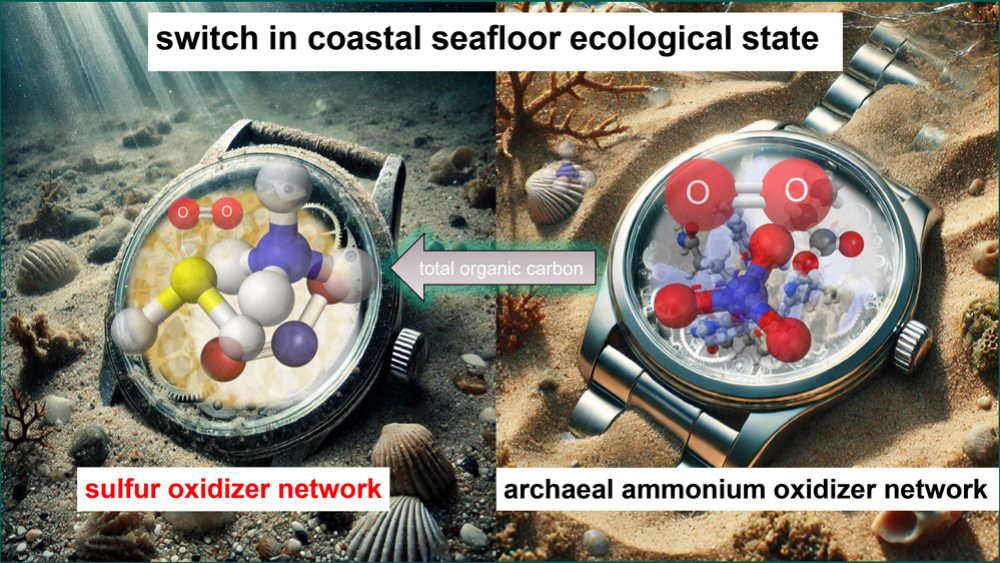

Recent evidence suggests that there is a major switch in coastal seafloor microbial ecology already at a mildly deteriorated macrofaunal state. This knowledge is of critical value in the management and conservation of the coastal seafloor. We therefore aimed to determine the relationships between seafloor microbiota and macrofauna on a regional scale. We compared prokaryote, macrofauna, chemical, and geographical data from 1546 seafloor samples, which varied in their exposure to aquaculture activities along the Norwegian and Icelandic coasts. We found that the seafloor samples contained either a network centralized by a sulfur oxidizer (42.4% of samples, *n* = 656) or a network centralized by an archaeal ammonium oxidizer (44.0% of samples, *n* = 681). Very few samples contained neither network (9.8% of samples, *n* = 151) or both (3.8% of samples, *n* = 58). Samples with a sulfur oxidizer network had a 10-fold higher risk of macrofauna loss (odds ratios, 95% CI: 9.5 to 15.6), while those with an ammonium oxidizer network had a 10-fold lower risk (95% CI: 0.068 to 0.11). The sulfur oxidizer network was negatively correlated to distance from Norwegian aquaculture sites (Spearman rho = −0.42, *p* < 0.01) and was present in all Icelandic samples (*n* = 274). The ammonium oxidizer network was absent from Icelandic samples and positively correlated to distance from Norwegian aquaculture sites (Spearman rho = 0.67, *p* < 0.01). Based on 356 high-quality metagenome-assembled genomes (MAGs), we found that bicarbonate-dependent carbon fixation and low-affinity oxygen respiration were associated with the ammonium oxidizer network, while the sulfur oxidizer network was associated with ammonium retention, sulfur metabolism, and high-affinity oxygen respiration. In conclusion, our findings highlight the critical roles of microbial networks centralized by sulfur and ammonium oxidizers in mild macrofauna deterioration, which should be included as an essential part of seafloor surveillance.

## Abstract 2

Seafloor microbial networks driven by sulfur or ammonium oxidizers strongly influence macrofauna health, emphasizing their role in coastal management.

## Introduction

The coastal seafloor represents one of the richest and most productive marine ecosystems.^1^ The seafloor is an integral component of ocean ecosystems, providing a habitat for biological diversity as well as essential services such as nutrient recycling and production of trace elements.^2^ Despite the large geographical area encompassed by the coastal seafloor, the factors influencing seafloor biodiversity and ecosystem function are poorly understood.^3,4^ This lack of knowledge is critical as the coastal seafloor can be a recipient for anthropogenic waste, such as that from aquaculture farms,^5^ sewage,^6^ and industry.^7^ The association to organic carbon remains particularly enigmatic. We have recently found that organic carbon is correlated with decreased prokaryote diversity,^8^ which contrasts with other recent studies which showed increased prokaryote diversity for high organic carbon loads.^9,10^ In our study, we found that the low-diversity communities for high organic carbon were associated with the sulfur-oxidizing genus *Sulfurovum*, while the high-diversity communities for low organic carbon were associated with the ammonium-oxidizing archaeon *Nitrosopumilus*.^8^

*Nitrosopumilus* is one of the most abundant microorganisms in marine environments.^2,11,12^*Nitrosopumilus* occupy oligotrophic environments utilizing ammonium as an energy source and bicarbonate as a source for carbon.^12^ In sediments, *Nitrosopumilus* has also been proposed to utilize organic carbon.^13^*Nitrosopumilus* is proposed as an important ecological service provider through vitamin production.^14,15^ It has also been estimated that dark carbon fixation by *Nitrospumilus* provides about 4% of the organic carbon in marine ecosystems.^16^

*Sulfurovum*, on the other hand, has primarily been associated with geothermal, rather than human, activities,^17^ although there are some reports of noteworthy abundances related to dredged sediments and fish farms.^18^ A high level of *Sulfurovum* has also been identified in the brine of the hypersaline Antarctic Lake Vida.^19^ The lake brine contained high levels of carbon dioxide, ammonium, nitrate, and DMSO, while sulfides were under the detection limit. This suggests that *Sulfurovum* can be selected under quite extreme conditions, despite being nonsulfidic.

For the macrofauna, most knowledge about the effect of human activity on seafloor diversity is derived from impact studies of aquaculture.^5,20^ To facilitate comparisons across geographic regions, various diversity indices have been developed. Some indices utilize eDNA-derived microorganism counts^21^ in a manner comparable to the well-established macrofauna indices,^22^ wherein organisms are categorized into eco-groups based on their relative sensitivity to waste, such as for the AZTI’s Marine Biotic Index (AMBI).^23^ In AMBI, the macrofauna is divided into five classes, ranging from species very sensitive to organic enrichment (tolerance group 0) to first-order opportunist deposit-feeding species (tolerance group 6).

The macrofaunal indices, however, are generally not harmonized between countries.^24^ In Norway and Iceland, the most widely used macrofaunal index is the normalized ecological quality ratio (nEQR).^25^ nEQR is a measure of the level of ecological status that integrates information about diversity and key macrofauna taxa. The nEQR index ranges from 0 to 1, with 0 to 0.2 being severely deteriorated, 0.2 to 0.4 deteriorated, 0.4 to 0.6 moderately deteriorated, 0.6 to 0.8 mildly deteriorated, and 0.8 to 1 representing the macrofaunal natural state.^26^

For highly deteriorated macrofauna, it is well established that anoxia and sulfides are drivers of deterioration.^27^ We lack, however, a good understanding of mechanisms underlying the low- to nondeteriorated macrofauna. Within benthic assemblages, it is likely that microorganisms provide essential nutrients or other exogenous compounds required by macrofauna.^28,29^ Alterations of key microbial service providers could therefore lead to major unforeseen effects on the macrofauna.^30^

This work aimed to identify prokaryotic taxa and processes associated with the macrofaunal ecological status at a regional scale, focusing on samples with a high ecological state. This was achieved through large-scale comparative studies, including prokaryote metabarcoding, macrofaunal assessment, metagenomic analyses, and a range of chemical measurements of more than 1500 samples along the Norwegian and Icelandic coasts, with varying exposure to aquaculture activities.

## Materials and Methods

### Sample Collection

The 1546 sediment samples included in our work were collected at 41 different sites along the Norwegian coastline, which spans three main bodies of water, the Barents Sea, Norwegian Sea, and the North Sea, and 9 different sites along the western Icelandic coast. The samples were collected from 2021 to 2023 during the summer half of the year, from March to September. For the purposes of this study, samples which were collected in the coastal zones and within fjords are referred to in three latitudinal groups according to those water bodies. Most of the samples were collected from aquaculture sites, but other samples were also included, such as those sampled prior to the establishment of aquaculture farms. An overview of the samples included is provided in Table S1 and in the Supporting Information Metadata.

From each station, two Van Veen grabs (0.1 m^2^) were collected, one intended for chemical analyses and the other for macrofauna. According to requirements by Norwegian authorities as specified in Norwegian standard NS 9410, the chemical samples were conducted for the upper 1 cm of the sediment, while total organic matter (TOM) and grain size were collected from the upper 5 cm. eDNA samples were also gathered from the upper 1 cm surface of the macrofauna grabs. Sediments from the macrofauna grabs were sieved through a sieve with a 1 mm mesh size, preserved, and analyzed according to NS9410 and ISO 16665:2014 using the normalized ecological quality ratio (nEQR) for assessing the ecological state of the macrofauna.^25,26^ Information about the AZTI’s Marine Biotic Index (AMBI) sensitivity index for each taxon was derived from Rygg and Norling.^31^ Briefly, the total organic carbon (TOC) was calculated based on weight loss after combustion at 495 °C for dried sediments. Total *N* as determined using ISO 13395:1996, representing combined nitrate and nitrite nitrogen measurement. Copper and zinc were measured using inductively coupled plasma mass spectrometry (ICP-MS) after acid solubilization.

### eDNA Sampling and Extraction

The sampling for eDNA was done from the grab intended for chemical analyses. Sample material was collected from the upper 1 cm of the undisturbed sediment surface by inserting a spoon through the inspection hatch of the grab. The sediment samples were frozen at −20 °C for shipment and further processing in the laboratory, as previously described.^8^

All eDNA extractions were done using a KingFisher Flex automated extraction platform (Thermo Scientific, USA), with the magnetic bead-based MagAttract PowerSoil KF kit (QIAGEN, Germany). 250 μL from each of the homogenized sediment samples was added to the bead plate, and we did a mechanical lysis for 4 × 30 s at 1800 rpm on a FastPrep 96 (MP Biomedicals, USA). All the centrifuge steps were done for 21 min at 1300*g* on a plate centrifuge, PlateSpin II (KUBOTA, Japan). The rest of the extraction was done following the manufacturer’s protocol.

Each plate contained a mock community, working as a positive control under the extraction and further processing of the samples.

### PCR Amplification and 16S rRNA Gene Library Preparation

The V3–V4 region of the 16S rRNA gene was amplified using 0.2 μM of the primers PRK341 forward (5′-CCTACGGGRBGCASCAG-3′) and PRK806 reverse (5′-GGACTACYVGGGTATCTAAT-3′),^32^ together with 1× HOT FIREPol Blend Master Mix Ready to Load (Solis BioDyne, Estonia). The following amplification program was used: 95 °C for 15 min, 25 cycles of 95 °C for 30 s, 55 °C for 30 s, and 72 °C for 45 s, followed by 72 °C for 7 min.

To purify the PCR products, we used 1× volume of Sera Mag Beads (Supplier). Amplicon purification was performed on Biomek 3000 or 4000 (Beckman Coulter, USA) automated liquid handling platforms, following the manufacturer’s protocol. The program for PCR purification was the same on both instruments. Some plates were also done manually due to technical issues, following the same protocol as on the instruments.

To index the amplicons, 1× FIREPol Master Mix Ready to Load (Solis BioDyne, Estonia) was used together with a combination of 16 forward and 36 reverse primers with Illumina indexes. The following PCR program was used: 95 °C for 5 min, 10 cycles of 95 °C for 30 s, 55 °C for 1 min, and 72 °C for 45 s, followed by 72 °C for 7 min.

To concentration-normalize and pool all the samples to one library, we quantified the samples using a VarioSkan LUX Multimode Microplate Reader (ThermoFisher Scientific, USA) to measure the relative fluorescence unit (RFU) in each sample at a 260 nm wavelength. We then made a standard curve from measured ng/μL using a Qubit fluorometer and the dsDNA high-sensitivity assay kit. This standard curve was used to estimate the concentration in ng/μL in each sample, which was used in further calculations for normalization. In order to get a deep sequencing, due to the high diversity of the sediment samples, each library consisted of approximately 288 samples, which resulted in an average of 33 thousand sequencing reads per sample after filtering and processing. A purified library was sent to the Norwegian Sequencing Centre in Oslo and sequenced on an Illumina MiSeq v3 platform.

### Whole Genome Shotgun Sequencing

To perform a whole genome shotgun sequencing on a selection of 94 sediment samples, we used the Illumina Nextera DNA Flex Library Prep. The input of genomic DNA was between 10 and 49 ng, and we followed the manufacturer’s protocol according to the amount of DNA. The index adapters we used were the Nextera DNA UD Indexes, set C (IDT for illumina, USA), and during the library cleanup, we followed the protocol for small PCR fragments (<500 bp). The finished library was sequenced on a NovaSeq 6000 PE150 at Novogene UK with an average sequencing depth of 132 million paired reads per sample.

### Data Processing, Assignments of Taxonomy and Function

The 16S rRNA gene raw data was demultiplexed and then processed using the VSEARCH software version 2.22.1.^33^ Reads were trimmed (3′ end, 20 bases off R1 and 60 off R2 reads), merged, and filtered by the fastq quality scores (mean error probability < 0.01). Dereplicated reads were clustered into OTUs at 95% identity and checked for chimeras. The 95% level was chosen because of the large size of the data set and the high diversity of the samples. A table of read counts for all OTUs in all samples was computed. The OTU sequences were assigned a taxonomic classification using the SINTAX algorithm^34^ implemented in VSEARCH, with the RDP database.^35^ No threshold was used for the taxonomic assignment. Functional assignments for the 16S rRNA gene data were done using the FAPROTAV 1.2.7 database.^36^

The reads from the whole genome sequencing were trimmed, filtered, and merged (if possible) using the Bbmap software version 39.01 (sourceforge.net/projects/bbmap/). Each sample was assembled using SPAdes version 3.15.5^37^ in “meta” mode. Coverages for the resulting contigs in each sample were computed by CoverM version 0.6.1 (github.com/wwood/CoverM), and the contigs were binned using both MaxBin2 version 2.2.7^38^ and MetaBat2 version 2.15.^39^ All bins were given quality scores (completeness and contamination) with CheckM2 version 1.0.1^40^ and then dereplicated with dRep version 3.4.0.^41^ Dereplicated bins with completeness at least 75% and contamination below 25% were considered metagenome-assembled genomes (MAGs), and these were given a taxonomic assignment with GTDBTk version 2.3.2^42^ using the GTDB version 2.07 database. We also computed the mean coverage for all MAGs in all samples, again using CoverM, and finally, all MAGs were given functional annotations with DRAM version 1.4.^43^

### Statistical and Ecological Analyses

All statistical analyses were done using the implementations in the MATLAB R2023 programming environment. We mostly used preprogrammed functions that can be run in MATLAB. For the analyses involving pairwise correlations, we used the “corr” function with Spearman correlation. Nonparametric tests were used to avoid the linearity assumption. To determine differences between groups of continuous variables, we used the “kruskalwallis” function, which implements the nonparametric Kruskal–Wallis test. The distribution of categorical variables was investigated using the Chi-Square Test of Independence; this test is implemented in the MATLAB “crosstab” function. All *p*-values were corrected for false discovery rate (FDR) using the “mafdr” function with the Benjamini and Hochberg correction.^44^

Modeling of nonlinear associations was done using cross-validated generalized additive models (GAMs) with the function “fitgram” using the following parameters: “CrossVal”, “on”, “KFold”, 10, and “FitStandardDeviation”, true. The function was also run without cross-validation for plotting purposes. The partial dependences were plotted using the “plotPartialDependence” function, with the following parameters: “Conditional” and “absolute”. Due to the relatively large differences in the microbiota composition across all individual sediment samples,^45^ we did not take into account the hierarchical structure of the data in the GAM modeling.

The alpha diversity analyses were done using an in-house MATLAB implementation of the Simpson’s *D* and Shannon H indexes. For the 1 – Simpson’s *D* index, we used the following implementation: 1 – *D* = 1 – ∑(*pi*)^2^, where *pi* is the portion of a specific OTU. For Shannon *H*, we used the following implementation: *H* = −∑(*pi* × ln *pi*). The beta-diversity analyses were conducted using the MATLAB Fathom package (www.usf.edu/marine-science/research/matlab-resources/fathom-toolbox-for-matlab.aspx). A Bray–Curtis distance matrix was generated using the Fathom function “f_dis” with the “bc” parameter. The “f_dis” distance matrix was then used as input in the “f_pcoa” function, which performs a principal coordinates analysis (PCoA). The output from the “f_pcoa” function was finally used as an input in the “f_pcoaPlot” function, generating a graphical representation of the PCoA plot.

Binarization of the 16S rRNA gene sequence data was based on “hartigansdipsigniftest”, with 10,000 bootstrap replicates. This test determines the deviation from a unimodal distribution, as outlined by Hartigan.^46^ If a statistically significant deviation from unimodality was identified (*p* < 0.05), then a threshold was set to binarize the data. Odds ratios (ORs) were determined for categorical data. Macrofauna data were binarized, assuming that nEQR values below 0.8 represent deteriorated ecological states, while values above 0.8 represent healthy states. The exposure was represented by the presence of the networks of microorganisms (*Nitrosopumilus* and *Sulfurovum*). The odds ratios were calculated by dividing the relative occurrence of macrofauna loss (or other categorical data) in the presence of the respective group of microorganisms by the relative occurrence in the absence of the group of microorganisms. The 95% confidence intervals were calculated as described by Altman.^47^

## Results

### Correlation Networks Reveal a Strong Negative Correlation between the Genera *Nitrosopumilus* and *Sulfurovum*

We identified a total of 49,543 prokaryote 16S rRNA gene OTUs (clustering at 95% similarity) representing 2387 genera and 61 phyla, with 26 phyla showing a mean abundance above 0.1%. Proteobacteria was clearly the most abundant phylum, with 51.6% of the sequencing reads, 972 of the genera, and 19,962 of the OTUs (Table 1).

**Table 1:** Summary of the Phyla with Coverage above 0.1% for the 16S rRNA Sequence Data

phylum	reads (%)	genera (#)	OTUs (#)
Proteobacteria	51.57	972	19,962
Actinobacteria	11.58	247	1349
Bacteroidetes	7.40	267	3106
Acidobacteria	6.02	52	2612
Campylobacterota	5.12	22	517
Cyanobacteria/Chloroplast	4.45	38	423
Firmicutes	2.80	505	3978
Chloroflexi	1.74	23	611
Nitrospirae	1.03	3	118
Verrucomicrobia	1.00	28	952
Planctomycetes	0.99	37	1445
Thaumarchaeota	0.82	2	33
Spirochaetes	0.80	17	611
Latescibacteria	0.57	1	345
Deferribacteres	0.35	10	188
Ignavibacteriae	0.27	2	56
BRC1	0.23	1	246
Candidatus_Saccharibacteria	0.21	1	187
Gemmatimonadetes	0.20	4	59
Fibrobacteres	0.18	3	252
Aminicenantes	0.13	2	82
Woesearchaeota	0.12	5	848
Rhodothermaeota	0.12	7	73
Armatimonadetes	0.11	8	274
Fusobacteria	0.11	9	32
Hydrogenedentes	0.11	1	212

To determine potential microbial interactions, we investigated the pairwise correlation structure for all the prokaryote genera identified. In total, we identified 174,743 moderate correlations with Spearman rho > 0.3 and 28,557 with rho < −0.3. More than 200 genera showed over 500 positive correlations, while only one genus, *Sulfurovum*, showed more than 500 negative correlations. The overall strongest pairwise negative correlation was identified between the genera *Nitrosopumilus* and *Sulfurovum* (Spearman rho = −0.79, *p* = 5 × 10^–324^).

In total, 25.2% of the genera showed opposite correlation patterns for *Nitrosopumilus* and *Sulfurovum*. A large subnetwork of 516 genera showed both moderate positive correlation with *Nitrosopumilus* (Spearman rho > 0.3) and moderate negative correlation with *Sulfurovum* (Spearman rho < −0.3), while 79 genera showed moderate positive correlation with *Sulfurovum* and moderate negative correlations with *Nitrosopumilus* (Figure S1).

**Figure 1:**
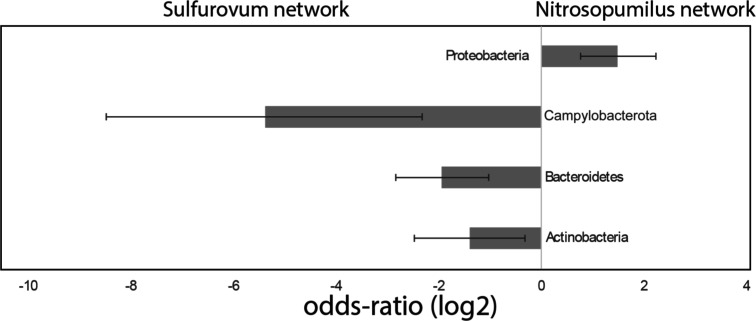
Odds ratio for genera within the *Sulfurovum* and *Nitrosopumilus* networks for 16S rRNA gene sequence data. The bars represent the odds ratio for the different phyla. Negative values represent overrepresentation for the *Sulfurovum* network, while positive values represent overrepresentation for the *Nitrosopumilus* network. Error bars represent the 95% confidence interval for the odds ratios.

The *Sulfurovum* network showed an overrepresentation of genera belonging to the phyla Campylobacteriota, Bacteroidetes, and Actinobacteria, while the *Nitrosopumilus* network showed an overrepresentation of genera belonging to Proteobacteria and Acidobacteria (Figure 1).

### Macrofaunal Ecological Status Can Be Predicted Based on the *Nitrosopumilus* and *Sulfurovum* Networks

**Figure 2:**
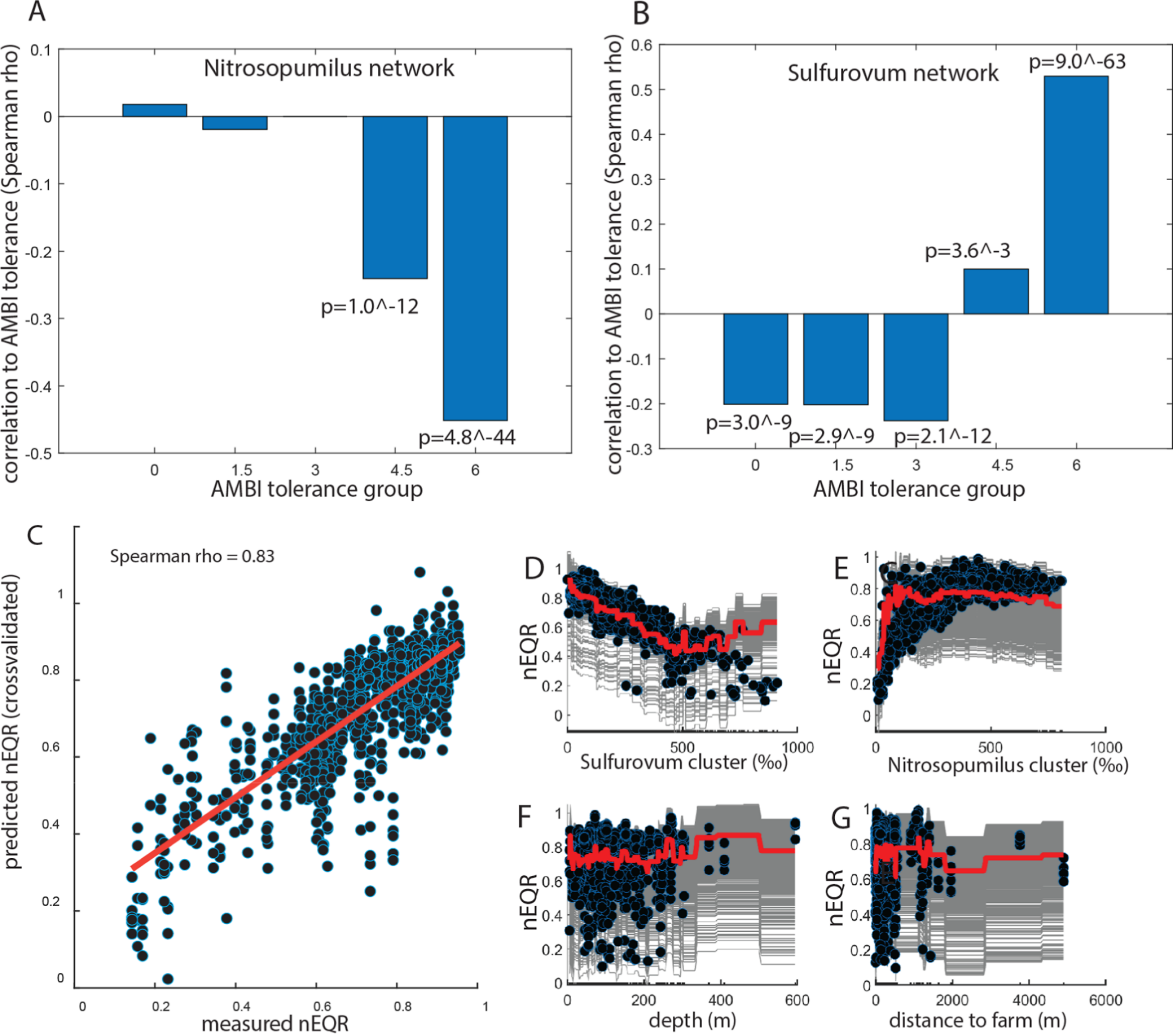
Model for the association between macrofauna and microbiota. (A) Correlations between AMBI tolerance groups and the *Nitrosopumilus* network. (B) Correlation between AMBI tolerance groups and the *Sulfurovum* network. (C) Correlation between measured nEQR and the cross-validated prediction using the GAM model. (D–F) The dots represent the actual measurements for each of the four components in the GAM model. Individual conditional expectation plots are presented as gray lines for each of the measurements, while the thick red line represents the partial dependence in the GAM model.

We identified 407 macrofauna taxa, which were classified with AZTI’s Marine Biotic Index (AMBI) tolerance groups.^31^ For the *Nitrosopumilus* network, we identified a negative correlation to the macrofauna taxa belonging to the AMBI high tolerance group (Figure 2A). For the *Sulfurovum* network, we identified a negative correlation toward groups with low AMBI tolerance, while for the macrofauna groups with high tolerance, we identified a strong positive correlation (Figure 2B).

We used cross-validated generalized additive models (GAMs) to determine the association of the *Nitrosopumilus* and *Sulfurovum* networks toward the overall ecological state, as represented by the nEQR values for the macrofauna. We found that GAM-predicted nEQR values correlated very well with the observed values (Figure 2C). In the model, the relative abundance of both the *Sulfurovum* and *Nitrosopumilus* networks showed nonlinear partial dependence to the nEQR values (Figure 2D,E). For the *Sulfurovum* network, there was a negative linear trend in the partial dependence to a nEQR value of about 0.4 (Figure 2D). The relationship between nEQR and the relative content of the *Nitrosopumilus* network showed a threshold at about 0.2, as visualized in the partial dependence plot (Figure 2E). Depth and distance from aquaculture sites showed low influence in the model, as indicated by the flat curves for the partial dependencies (Figure 2F,G).

### *Nitrosopumilus* and *Sulfurovum* Networks Showed Opposite Associations to Chemical and Physiochemical Composition

**Figure 3:**
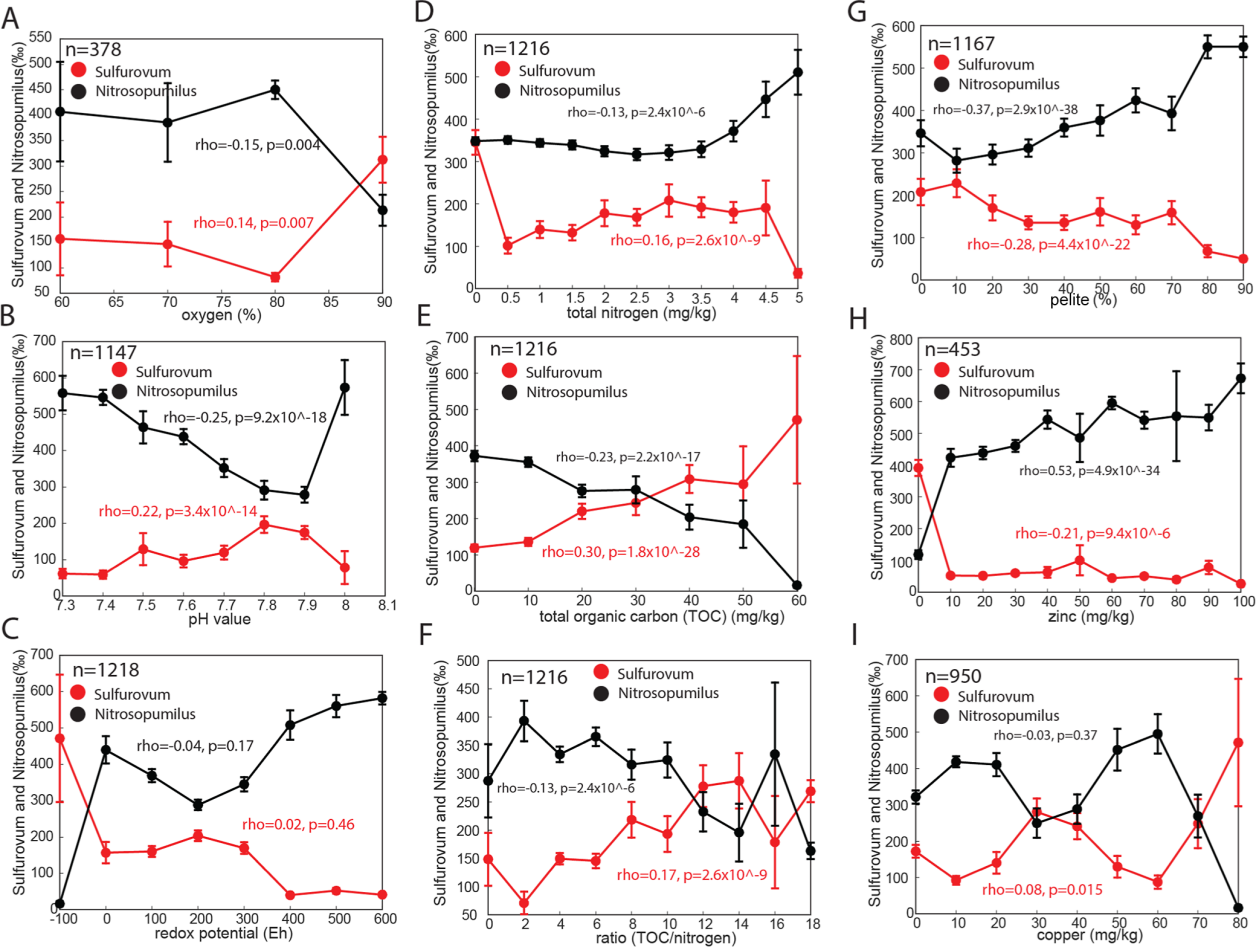
Chemical associations of the *Sulfurovum* and *Nitrosopumilus* networks. The panels illustrate the direct association between the *Sulfurovum* and *Nitrosopumilus* network clusters.

The chemical measurements unveiled several strong correlations with prokaryote community composition (Figure 3). The *Sulfurovum* network was weakly positively correlated, while the *Nitrosopumilus* network was weakly negatively correlated to oxygen saturation above 80% (Figure 3A). The *Nitrosopumilus* network showed a strong negative correlation with pH up to 7.9, with a switch to a strong positive correlation to pH greater than 7.9 (Figure 3B), while the *Sulfurovum* network showed a positive correlation to pH across the observed pH scale (Figure 3B). For redox potential, there was no clear correlation of either network (Figure 3C). There was a slight negative correlation between the *Nitrosopumilus* network and total nitrogen for values <3.5 mg/kg, above which a positive correlation was observed. The *Sulfurovum* network showed the opposite correlative trend with total nitrogen (Figure 3D). A strong negative correlation to organic carbon was shown for the *Nitrosopumilus* network, while the *Sulfurovum* network showed a positive correlation (Figure 3E). At a ratio between organic carbon and nitrogen below 10, the *Nitrosopumilus* network was overrepresented in comparison to the *Sulfurovum* network (Figure 3F). The *Nitrosopumilus* network had a strong positive correlation to pelite, while the *Sulfurovum* network was negatively correlated (Figure 3G). Furthermore, there was a positive correlation between zinc and the *Nitrosopumilus* network, while correlation to zinc was negative for the *Sulfurovum* network (Figure 3H). Finally, copper showed a multimodal distribution, with a dip for the *Nitrosopumilus* network and a peak for the *Sulfurovum* network between 30 and 50 mg/kg (Figure 3I).

### Bimodal Distribution of *Nitrosopumilus* and *Sulfurovum* Networks

The *Nitrosopumilus* network displayed an apparent bimodal distribution pattern among sediment samples, with two overlapping peaks (Figure S2A), as evidenced by the Hartigan dip test of multimodality with a dip statistic of 0.052 and a *p*-value of 5.7 × 10^–4^. A minimum value for the dip was reached at approximately ∼330‰. On the other hand, *Sulfurovum* exhibited a dip statistic of 0.06 and a *p*-value of less than 1 × 10^–5^, with the first peak ending around ∼100‰ (Figure S2B).

**Figure 4:**
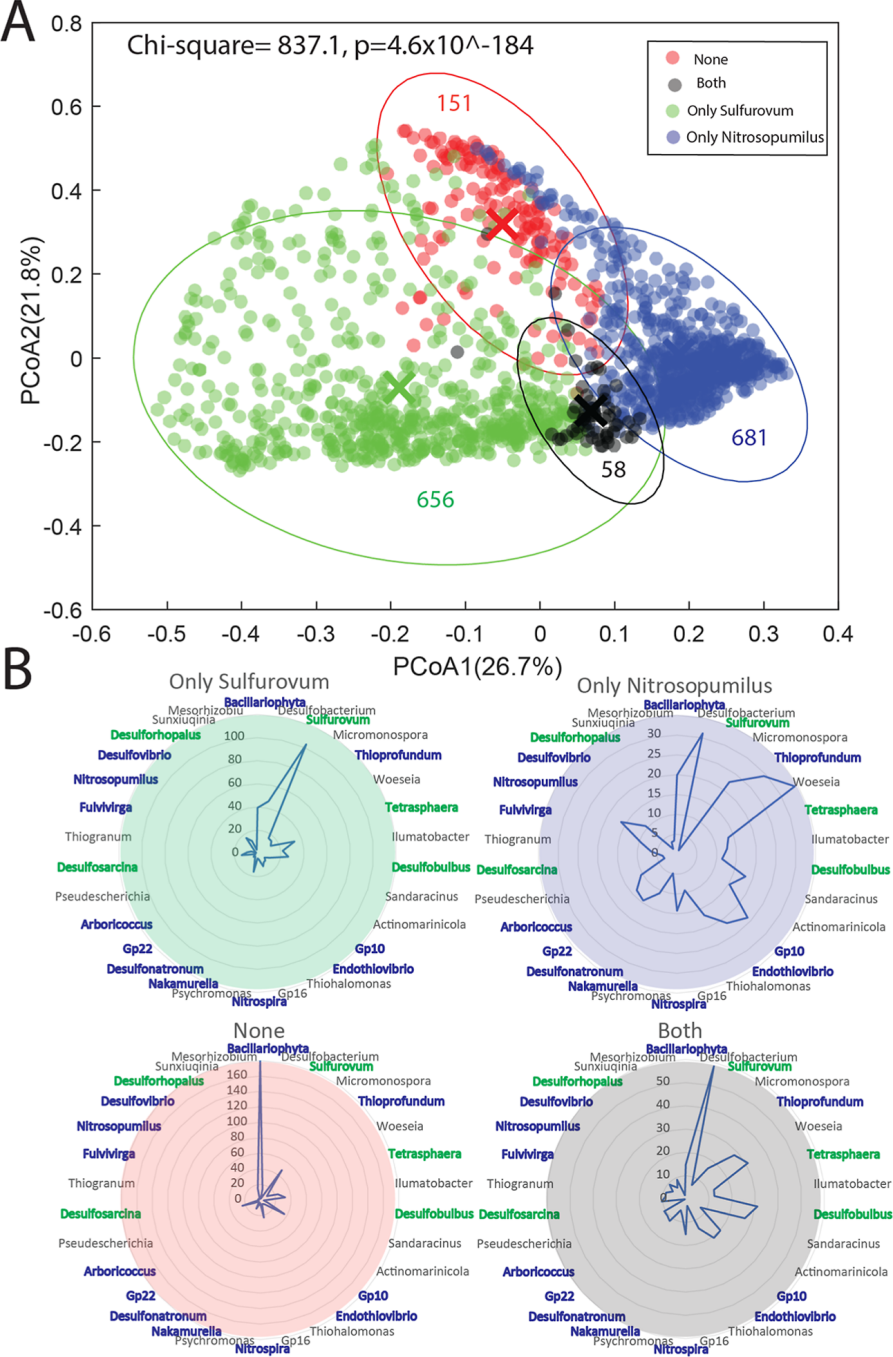
Taxonomic composition for the *Sulfurovum* and *Nitrosopumilus* network clusters. (A) PCoA plot illustrating the overall beta diversity for the samples containing the *Sulfurovum* and *Nitrosopumilus* network clusters. (B) Mean distribution (‰) of the dominating genera within the different networks. The genera are color-coded with blue if they belong to the *Nitrosopumilus* network cluster and green if they belong to the *Sulfurovum* network cluster. The ellipses represent the 95% confidence interval, while the crosses represent the centroids.

Based on the dip statistics, we converted the data to binary format at 330‰ for the *Nitrosopumilus* network and at 100‰ for the *Sulfurovum* network. The binary data was then visualized in a PCoA plot based on Bray–Curtis distances. This analysis revealed that the *Nitrosopumilus* network cluster is notably more compact compared to the *Sulfurovum* network cluster, which exhibited higher dispersion (Figure 4A). The samples containing both the *Nitrosopumilus* and the *Sulfurovum* networks were located at the intersection between the two clusters along the first PCoA axis.

The genus-level composition showed that *Sulfurovum* dominated in sediments whose prokaryote communities were dominated by OTUs associated with the *Sulfurovum* network. There was a larger range of genera that were observed to be abundant in sediments dominated by OTUs associated with the *Nitrosopumilus* network. Samples with ambiguous presence of both *Nitrosopumilus* and *Sulfurovum* networks showed a high relative abundance of chloroplasts from Bacillariophyta, while the intersect network contained a mix of *Sulfurovum*- and *Nitrosopumilus*-associated genera (Figure 4B).

### Association of Binarized Data with the Ecological State, Geography, and Investigation Type

To determine the risk of a mildly deteriorated ecological state (nEQR < 0.8) for samples classified as rich in OTUs associated with either the *Nitrosopumilus* or the *Sulfurovum* networks, we calculated the respective odds ratios (ORs). Sediments classified as rich in the *Sulfurovum* network had greater than a 10-fold increase in risk of a deteriorated ecological state, with nEQR values below 0.8 (95% CI: OR 9.5 to 15.6). In contrast, sediments classified as rich in the *Nitrosopumilus* network showed a 10-fold decreased risk of ecological state deterioration (95% CI: OR 0.068 to 0.11).

**Figure 5:**
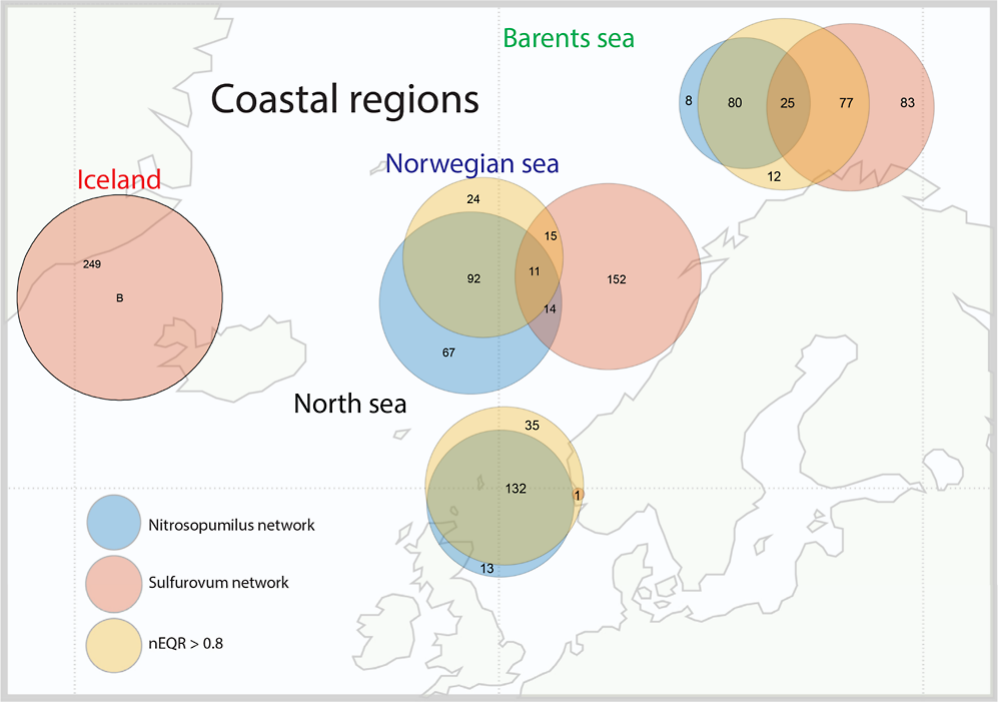
Geographical distribution of *Sulfurovum* and *Nitrosopumilus* network clusters. The Venn diagrams illustrate the association between the *Nitrosopumilus* and *Sulfurovum* network clusters and high nEQR values (>0.8).

The geographical distribution showed a very clear east–west gradient. All the Icelandic sediments were classified as rich in the *Sulfurovum* network, with no sediment samples having a particularly good ecological state with nEQR values above 0.8. On the other hand, for the samples from the North Sea, we found a higher prevalence of sediment samples classified as rich in the *Nitrosopumilus* network coincided with particularly good (nEQR > 0.8) ecological state classification. There was also a shift toward more sediment samples classified as *Sulfurovum*-enriched further north in Norway (Figure 5).

Pre-examinations showed a clear association with nEQR values above 0.8. Pre-examinations were also positively associated with the *Nitrosopumilus* network and negatively associated with the *Sulfurovum* network. The other examinations included showed opposite association patterns than that for the pre-examinations (Table S2).

### Metabolic Potential Showed Distinct Functionalities between the *Nitrosopumilus* and *Sulfurovum* Networks

**Figure 6:**
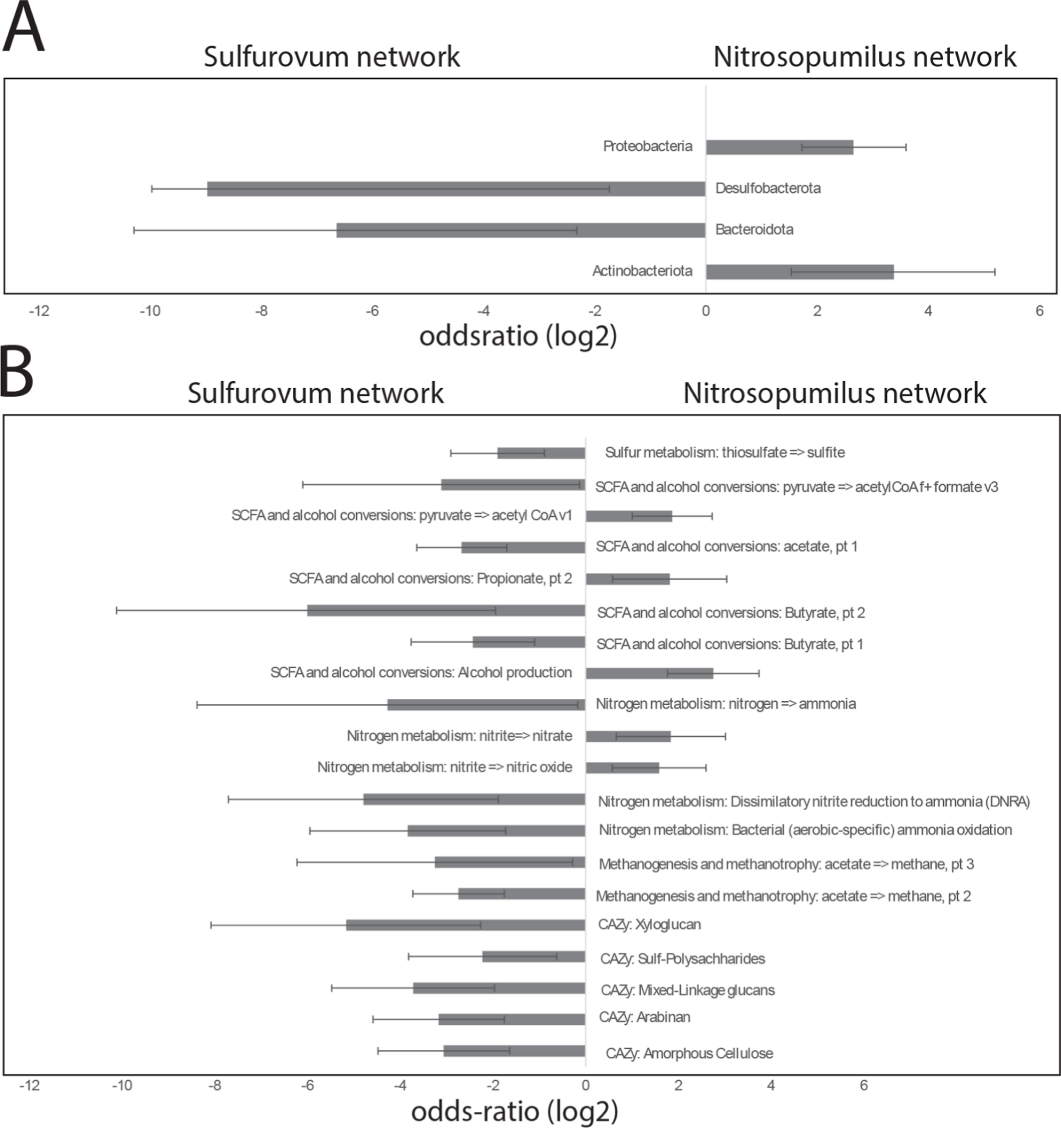
Odds ratios for the distribution between the *Sulfurovum* and the *Nitrosopumilus* networks for MAGs. (A) Phyla with statistically significant distribution between the *Sulfurovum* and the *Nitrosopumilus* networks. (B) Metabolic functions with a statistically significant difference in distribution between the *Sulfurovum* and *Nitrosopumilus* networks. The filled bars represent odds ratio with respect to the *Sulfurovum* and *Nitrosopumilus* networks, with the error bars representing the 95% confidence interval for the odds ratios.

Functional investigations were conducted based on a catalogue of 356 metagenome-assembled genomes (MAGs) obtained by deep shotgun sequencing of 94 of the samples used for metabarcoding analysis above (shotgun-sequenced samples are marked in the Supporting Information Metadata). The taxonomic distribution of the MAGs was comparable to that of 16S rRNA gene data, with dominance of Proteobacteria and Actinobacteria/Actinobacteriota (Table 2; Supporting Information MAGinfo). We also observed similar patterns as for the 16S rRNA gene data in the distribution between the *Sulfurovum* and the *Nitrosopumilus* networks for Proteobacteria and Bacteroidota, with Proteobacteria being associated with the *Nitrosopumilus* network and Bacteroidota with the *Sulfurovum* network (Figure 6A). A direct taxonomic comparison between 16S and shotgun data, however, is difficult as RDP taxonomy was used for the 16S rRNA gene sequence data, while the GTDB taxonomy was used for the shotgun data.

**Table 2:** Summary of the Phyla with Abundance above 0.1% for the MAG Sequence Data

phylum	coverage (%)	genera (#)	MAGs (#)
Proteobacteria	37.36	49	132
Actinobacteriota	16.45	8	35
Thermoproteota	15.22	5	13
Myxococcota	9.72	2	10
Desulfobacterota	5.80	17	41
Acidobacteriota	4.07	4	18
Nitrospirota	3.85	2	4
Nitrospinota	3.18	1	1
Bacteroidota	1.84	23	50
Planctomycetota	0.56	4	7
Desulfobacterota_D	0.32	1	2
Campylobacterota	0.28	5	12
Methylomirabilota	0.21	1	1
Firmicutes	0.21	2	4
Myxococcota_A	0.19	1	2
Schekmanbacteria	0.15	1	1
BMS3Abin14	0.12	1	1
Verrucomicrobiota	0.10	2	2

The reconstructed metabolic data showed distinct differences in carbon fixation strategies and respiratory chains between the *Nitrosopumilus* and *Sulfurovum* networks (Table 3). *Nitrosopumilus* showed an overrepresentation of MAGs with the 3-hydroxypropionate bicycle (3-HPA), which utilizes bicarbonate for carbon fixation. Conversely, the *Sulfurovum* network showed an overrepresentation of the acetyl-CoA pathway, utilizing carbon dioxide for fixation. For the respiratory chains, the *Sulfurovum* network showed an overrepresentation of high-affinity complexes like Complex III (cytochrome bd ubiquinol oxidase) and Complex IV (high-affinity cytochrome bd ubiquinol oxidase). In contrast, the *Nitrosopumilus* network showed an overrepresentation of more low-affinity complexes, such as Complex IV (cytochrome *c* oxidase) and Complex III (cytochrome *bc*1 complex).

**Table 3:** Carbon Dioxide Fixation Pathways and Respiratory Complexes Derived from MAG Data

metabolic pathway/complex	*Nitrosopumilus* network^a^	*Sulfurovum* network^a^	*p*-value^b^
3-hydroxypropionate bicycle	0.107	0.007	<0.0001
acetyl-CoA pathway, CO2=>acetyl-CoA	0.000	0.066	<0.005
Complex IV low affinity: cytochrome c oxidase	0.120	0.054	<0.0001
Complex II: Fumarate reductase, prokaryotes	0.198	0.033	<0.195
Complex I: NADH dehydrogenase (ubiquinone) 1 alpha subcomplex	0.020	0.000	<0.0005
Complex III: cytochrome *bd* ubiquinol oxidase	0.110	0.298	<0.0005
Complex IV high affinity: cytochrome *bd* ubiquinol oxidase	0.110	0.298	<0.0005
Complex III: cytochrome *bc*1 complex	0.130	0.066	<0.005
Complex II: succinate dehydrogenase (ubiquinone)	0.012	0.000	<0.05
Complex IV low affinity: cytochrome *o* ubiquinol oxidase	0.043	0.000	<0.05

a Numbers represent mean completeness.

b p-values were determined using the FDR-corrected Kruskal–Wallis test.

The metabolic pathways that show distinct distribution between the *Sulfurovum* and *Nitrosopumilus* networks are shown in Figure 6B. The *Nitrosopumilus* network is enriched in propionate and alcohol production pathways, as well as nitrogen metabolism processes like nitrite to nitrate and nitrite to nitric oxide. In contrast, the *Sulfurovum* network is enriched in thiosulfate to sulfite conversion, SCFA conversions related to acetate, butyrate, and formate production, as well as methanogenesis pathways involving acetate to methane conversion.

We also analyzed functional potential through 16S rRNA gene information using the Faprotax database.^36^ These analyses showed that the *Nitrosopumilus* network exhibited a higher functional diversity than the *Sulfurovum* network, with 80 functions identified for the former, as compared to 36 for the latter (Table S3). For the most abundant functions (overall mean above 4%), fermentation, hydrogen oxidation, and sulfur respiration were overrepresented in the *Sulfurovum* network, while ureolysis, nitrogen fixation, and aromatic hydrocarbon degradation were overrepresented for the *Nitrosopumilus* network (Figure S3). There was also a range of low-abundant traits that were overrepresented for the *Nitrosopumilus* network. These included one carbon metabolism, ammonium oxidation, and cellulose and plastic degradation (Table S3).

## Discussion

We identified major differences in metabolic processes between the *Nitrosopumilus* and the *Sulfurovum* networks. We further identified a strong positive correlation between the *Sulfurovum* network and macrofauna that are tolerant toward environmental stressors, while the correlations were negative for the *Nitrosopumilus* network. This may indicate that the *Sulfurovum* network is supported by invasive conditions through environmental stressors, while the conditions supporting the *Nitrosopumilus* network represent the seafloor ecological ground state.^2,9^

The shift between *Nitrosopumilus* and *Sulfurovum* networks starts at a high ecological state, so it is unlikely to be directly caused by free sulfides as free sulfides are commonly associated with severe ecological degradation.^48^ However, there could be indirect effects of free sulfides as the macroorganisms that are stress-tolerant commonly detoxify sulfides by conversion to thiosulfate or other low-toxic sulfur compounds.^48^ DRAM analyses indicated that *Sulfurovum* has the metabolic potential for thiosulfate oxidation. In a lab experiment, we have also shown that thiosulfate selectively enriches *Sulfurovum*.^49^ Unfortunately, since the sulfur metabolic processes are relatively poorly described,^50^ it is difficult to determine which chemical components are the drivers of *Sulfurovum* enrichment in nature. For the *Nitrosopumilus* network, it is likely that trace amounts of ammonium are the main energy source, although there have also been suggestions that *Nitrosopumilus* can take up organic carbon.^13^ However, we believe that organic carbon is an unlikely energy source as the oligotrophic seafloor is carbon-limited.^51,52^

The idea of keystone taxa, although controversial, underscores the significant ecological roles certain taxa play.^53^ In the context of the current study, *Nitrosopumilus* and *Sulfurovum* are candidates for potential keystone genera in the northern coastal sea regions investigated here. This is both due to their centrality in correlation networks^54^ and the strong association with the macrofauna. In particular, *Nitrosopumilus* seems important as a keystone taxon in coordinating a large network of more than 500 positively correlated genera, being strongly associated with slightly to unperturbed ecological states based on macrofaunal assessments.

In our work, the association between the *Nitrosopumilus* network and the macrofauna demonstrated an apparent threshold, with relative abundance in metabarcoding data above ∼20% supporting a particularly good ecological state according to the GAM model. This may indicate that *Nitrosopumilus* provides essential components to the macrofauna. In the current work, we identified both cobalamin-dependent carbon dioxide fixation and low-affinity cytochrome oxidases as associated with the *Nitrosopumilus* network. Previous research has suggested *Nitrosopumilus* as an essential contributor of cobalamin,^15,28,55^ with cobalamin being essential to maintain macroscopic life and support carbon dioxide fixation.^29^ Cytochrome oxidase is a key enzyme in aerobic metabolism; low-affinity variants indicate oxic conditions in the sediments associated with the *Nitrosopumilus* network.^56^ Additionally, *Nitrosopumilus* shows strong correlations with over 500 other genera, likely offering other, yet unidentified, ecological services vital for the macrofauna in the sea.

We found that the *Sulfurovum* network was associated with a decline in the macrofauna ecological state and an increase in macrofauna that are tolerant to stressors.^23^ The *Sulfurovum* network showed a high prevalence of cytochrome *bd* oxidases. Cytochrome *bd* oxidases enable respiration at submicromolar concentrations and protect against toxic compounds such as sulfides.^57^ This may indicate that microbes in the *Sulfurovum* network are adapted to respiration at low oxygen concentrations, below what is tolerated by the nontolerant macrofauna.^48^

We observed a concordance between the 16S rRNA gene sequence functional inference and shotgun functional annotations with respect to the importance of sulfur respiration for the *Sulfurovum* network-associated microbes.^8,58^ For oxygen respiration, on the other hand, the shotgun data did unveil a unique distinction between the *Nitrosopumilus* and the *Sulfurovum* networks, which was not detected by 16S rRNA gene inference. Microbes within the *Sulfurovum* network seemed more efficient in respiration under low oxygen concentrations through cytochrome *bd* oxidases,^57^ as compared to microbes belonging to the *Nitrosopumilus* network. Furthermore, shotgun sequencing also unveiled a distinction in carbon dioxide fixation, with the *Nitrosopumilus* network having the ability of cobalamin-dependent utilization of bicarbonate through 3-HPA, while the *Sulfurovum* network showed a dependence on carbon dioxide through the acetyl-CoA pathway.^59^ The presence in the *Nitrosopumilus* network is consistent with low-affinity cytochromes since 3-HPA is the only carbon dioxide fixation cycle that is not sensitive to oxygen.^60^ The acetyl-CoA pathway, on the other hand, is associated with anoxic conditions, enabling carbon dioxide reduction by hydrogen.^61^ There was also a distinction for nitrogen metabolism, with the *Sulfurovum* network being associated with ammonification and carbohydrate metabolism, while the *Nitrosopumilus* network was associated with denitrification. Taken together, the shotgun data point toward distinction in some fundamental microbial processes that could have profound effects on ecosystems at the seafloor. Although there have been other efforts in seafloor shotgun sequencing, these have mainly been focused on special environments such as hydrothermal vents,^62^ while the knowledge from coastal seafloor microbial ecosystems is limited.^3^

We identified a strong east–west pattern with high *Sulfurovum* and low nEQR in the Icelandic samples. This pattern could be due to geothermal activity along the Atlantic Ridge, rather than human impact. Basalt from the Mid-Atlantic Ridge contains elevated levels of sulfides,^63^ which could be a driver for sulfur metabolism. Sulfur metabolism could also be the cause for the reduced ecological status observed around Iceland. However, there could also be other confounding factors, such as increased carbon dioxide and decreased pH.^64^

There was a surprising positive association between *Nitrosopumilus* and total nitrogen, while there was a negative association of this network with organic carbon. This may indicate a competitive advantage for *Nitrosopumilus* at low TOC/nitrogen ratios. A low ratio may prevent the outgrowth of heterotrophs since organic carbon is limiting. A high TOC/N ratio, on the other hand, promotes the *Sulfurovum* network. This is paradoxical as *Sulfurovum* is also an autotroph. One potential explanation, however, could be that high TOC/N ratios promote the release of reduced sulfur compounds,^65^ which *Sulfurovum* could oxidize.

Recent theories suggest that low-energy ecosystems select for high-diversity microbial networks.^66^ This could explain why the *Nitrosopumilus* network exhibits greater diversity than the *Sulfurovum* network as the latter is associated with energy-rich conditions through organic enrichment under oxic conditions. The high microbial diversity associated with organic carbon under anoxic conditions observed in other studies^9,10^ can also be explained by energy availability as anoxia represents a low-energy state.^67^

Surprisingly, zinc showed a strong positive association with the *Nitrosopumilus* network. This association could be due to the dependence of carbonic anhydrase on zinc for the catalysis of carbon dioxide to bicarbonate.^68^*Sulfurovum*, on the other hand, can utilize carbon dioxide directly without enzymatic conversion to bicarbonate.^69^ This may indicate that inhibition of bicarbonate formation through zinc limitation could provide a competitive advantage for *Sulfurovum*. Finally, we discovered a strong positive correlation between pelite and the *Nitrosopumilus* network. This may indicate that *Nitrosopumilus* thrives in regions with weak bottom currents, thus representing potential hotspots for supporting macrofauna diversity.^70^ Seafloor sites with weak bottom currents may also be more vulnerable to the accumulation of waste deposits due to higher deposition rates in comparison with sites with strong bottom current.^71^

A limitation with our study is the lack of a balanced design since the sampling was done in conjunction with other governmentally enforced studies. The apparent overrepresentation of high nEQR for the North Sea coastal regions could potentially be due to a bias in sampling design since samples were not collected from a representative balance of aquaculture impact. Balanced sampling design, among other aspects, should be addressed in more targeted studies. Despite the limitations, our data set represents a unique comparison of the microbiota and macrofauna over a wide geographical region. The relationships identified here provide a meaningful, function-based relationship between microbial networks and macrofauna that can be used for the purpose of environmental monitoring and management in the future.

## Data Availability

All code for the raw data processing is found in the compressed archive at https://arken.nmbu.no/~larssn/nitrosulf.tar.gzhttps://arken.nmbu.no/~larssn/nitrosulf.tar.gz. This archive contains a subfolder for code used for processing the 16S data, another subfolder for code used for processing WGS data, and a third subfolder with Apptainer containers. Scripting was done in R or UNIX shell, and any external software tools used are supplied as Apptainer containers. All processing of raw data was done on a high-performance computing cluster using a CentOS Linux 7.9 operating system. The raw 16S rRNA gene and shotgun sequencing data are available under the accession number PRJNA1128851 in the Sequence Read Archive (SRA).

## Supporting Information Available

Correlation networks, frequency data, inferred functional properties, sampling locations, association between investigation type and ecological state, and functional assignment with the Faprotax database (PDF)
MAG information (XLSX)
Metadata (XLSX)
Taxonomy data (XLSX)
