# Supplementary material for: Association of Microbial Networks with the Coastal Seafloor Macrofauna Ecological State
Source: Environ Sci Technol. 2025 Apr 11;59(15):7517–29. doi: 10.1021/acs.est.4c12464 (PMC12020364; doi:10.1021/acs.est.4c12464)
Supplement: Supplementary file 1 — es4c12464_si_001.pdf [file es4c12464_si_001.pdf]

Supporting Information for Publication

Association of microbial networks with coastal seafloor macrofauna ecological state

Tonje Nilsen Ragnhild Pettersen, Nigel Brian Keeley, Jessica Louise Ray, Sanna Majaneva, Morten Stokkan, Anja Hervik, Inga Leena Angell, Lars Gustav Snipen, Maud Ødegaard Sundt, and Knut Rudi

summary: 8 pages, 3 tables, 3 figures

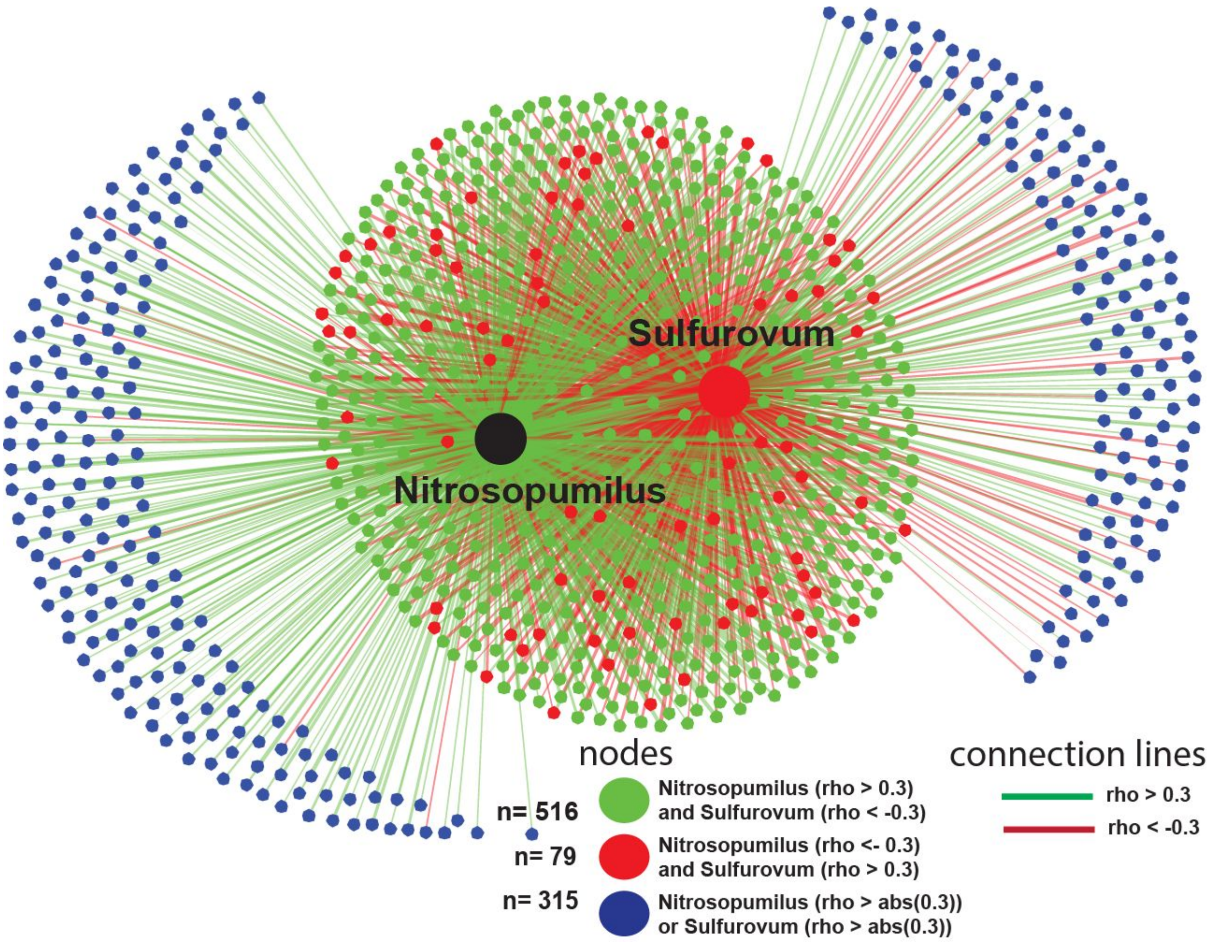

Suppl Figure 1 Correlation networks between *Sulfurovum* and *Nitrosopumilus*. The correlation network for *Nitrosopumilus* and *Sulfurovum* is illustrated by genera showing positive correlations with *Nitrosopumilus* and negative correlations with *Sulfurovum.* Each node represents a separate genus, while the genus *Nitrosopumilus* is highlighted in black, and the genus *Sulfurovum* in red. The color-code for the nodess represents the correlation patterns, as described in the figure. The color code for the connecting lines represents the direction correlation between the given pairs, with green lines representing Spearman rho > 0.3, while red lines represent rho < -0.3.

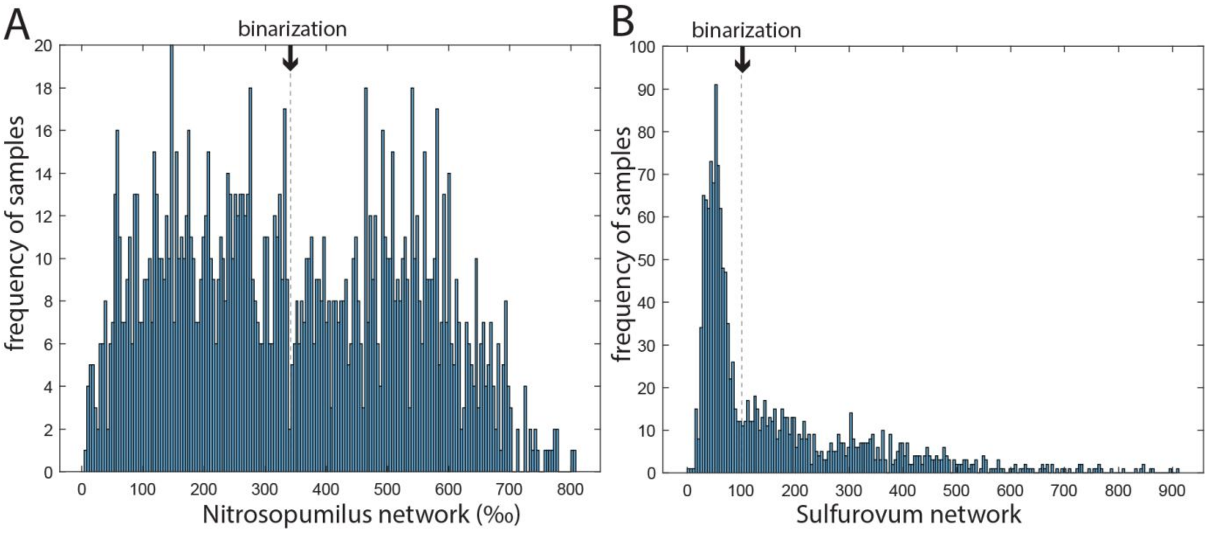

Suppl Figure 2. Frequency of (A) the *Nitrosopumilus* - and (B) the *Sulfurovum* network. The thresholds for binarization to the respective network clusters are marked.

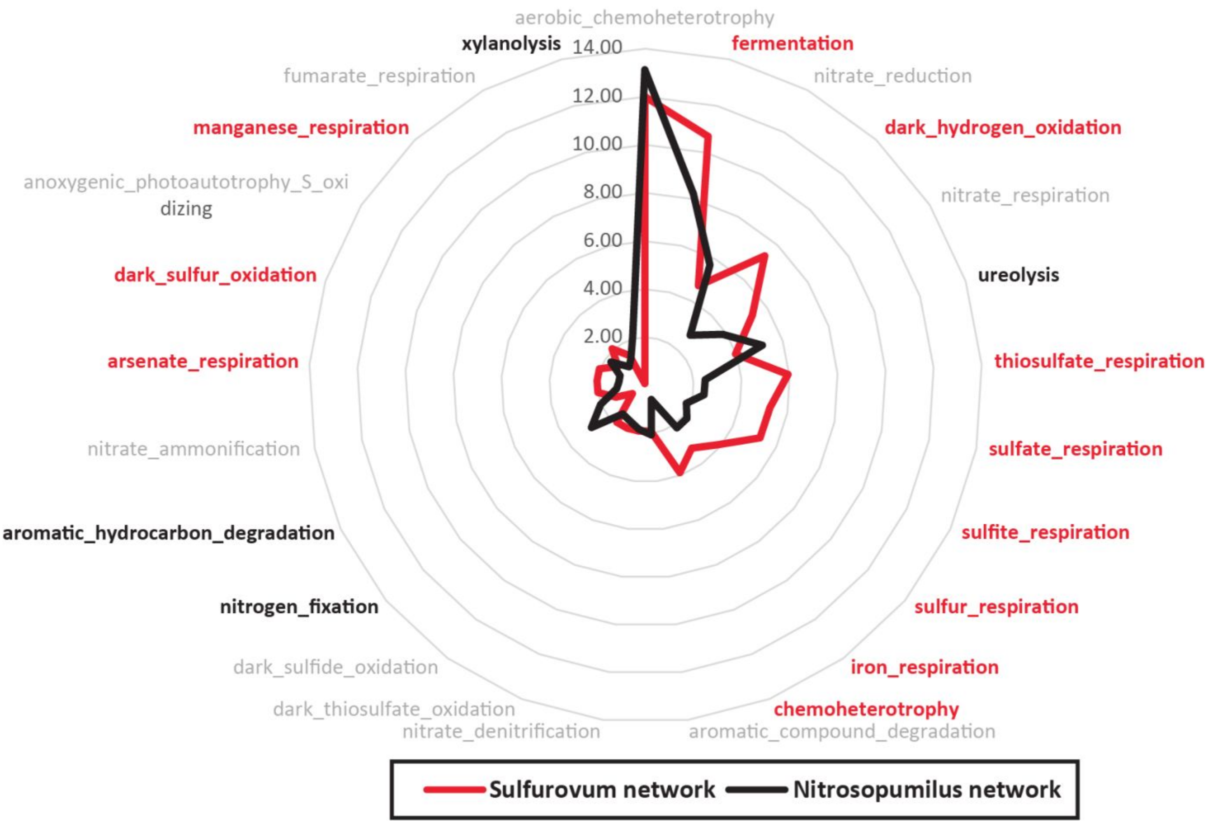

Suppl Figure 3. Inferred functional properties associated with the *Sulfurovum*- and the *Nitrosopumilus* network as determined from 16S rRNA gene analyses. The percentage of genera within the *Sulfurovum*- and *Nitrosopumilus* network with the Faprotax functional assignment are shown. Functions covering more than 2 percent of the genera are included. Functions are assigned to the respective networks, given an absolute log10 ratio above 0.1.

Suppl Table 1. Sampling locations

Country	Water body[1]	Examination	# Locations[2]	# Samples[3]
Norway	Norwegian Sea	Pre examination	5	165
Norway	Norwegian Sea	ASC-examination	3	96
Norway	Norwegian Sea	C-examination	5	210
Norway	Norwegian Sea	Recipient examination	1	60
Norway	Barents Sea	Pre examination	1	36
Norway	Barents Sea	C-examination	7	237
Norway	North Sea	Pre examination	11	312
Norway	North Sea	C-examination	2	30
Norway	North Sea	Recipient examination	2	12
Iceland	Danish Strait	Pre examination	1	30
Iceland	Danish Strait	C-examination	8	244

[1] Water body is defined as those with longitude below 0°east – Danish Strait, latitude below 60° north – North Sea, between 60° and 70° north – Norwegian Sea, and above 70° north – Barens sea.

[2] Four locations lacked geocoordinates. [3] One hundred and fourteen samples lacked geocoordinates.

Suppl Table 2 Association between investigation type and ecological state

Investigation type	nEQR		Nitrosopumilus network		Sulfurovum network	
	low (<0.8)	high (>0.8)	low (<33%)	high (>33%)	low (<10%)	high (>10%)
ASC-examination	96	0	67	29	23	73
C-examination	510	312	538	284	261	561
Pre-examination	123	432	153	402	490	65
Recipient examination	60	12	48	24	58	14

Suppl Table 3. Functional assignment with the Faprotax database (percentage)

function	*Sulfurovum*	*Nitrosopumilus*
aerobic_chemoheterotrophy	11.92	13.04
fermentation	10.60	8.13
nitrate_reduction	4.64	5.62
ureolysis	3.97	5.14
nitrate_respiration	5.30	3.83
nitrogen_fixation	0.66	2.87
dark_hydrogen_oxidation	7.28	2.75
thiosulfate_respiration	5.96	2.51
sulfate_respiration	5.30	2.51
sulfur_respiration	3.97	2.27
iron_respiration	3.31	2.27
aromatic_compound_degradation	1.99	2.15
aromatic_hydrocarbon_degradation	1.32	2.03
xylanolysis	0.00	2.03
sulfite_respiration	5.30	1.91
nitrate_denitrification	1.99	1.91
dark_thiosulfate_oxidation	1.99	1.67
anoxygenic_photoautotrophy_S_oxidizing	1.32	1.67
photoheterotrophy	0.00	1.67
dark_sulfide_oxidation	1.99	1.56
plastic_degradation	0.00	1.44
human_pathogens_all	0.66	1.32
nitrate_ammonification	1.99	1.20
fumarate_respiration	1.32	1.20
cellulolysis	0.66	1.20
dark_iron_oxidation	0.66	1.20
arsenate_respiration	1.99	1.08
dark_sulfur_oxidation	1.99	1.08
aerobic_anoxygenic_phototrophy	0.66	1.08
methanol_oxidation	0.00	1.08
nitrite_respiration	0.00	1.08
manganese_respiration	1.99	0.96
plant_pathogen	0.00	0.96
animal_parasites_or_symbionts	0.66	0.84
ligninolysis	0.00	0.84
anoxygenic_photoautotrophy_H2_oxidizing	0.00	0.84
chemoheterotrophy	3.97	0.72
manganese_oxidation	0.66	0.72
methanotrophy	0.00	0.72
human_gut	0.00	0.72
oil_bioremediation	0.00	0.72
intracellular_parasites	0.00	0.72
predatory_or_exoparasitic	0.00	0.72
arsenate_detoxification	0.66	0.60
invertebrate_parasites	0.66	0.60
aerobic_ammonia_oxidation	0.00	0.60

aliphatic_non_methane_hydrocarbon_degradation	0.00	0.60
dark_sulfite_oxidation	0.66	0.48
nitrous_oxide_denitrification	0.00	0.48
hydrocarbon_degradation	0.00	0.48
dark_oxidation_of_sulfur_compounds	0.66	0.36
methylotrophy	0.00	0.36
aerobic_nitrite_oxidation	0.00	0.36
arsenite_oxidation_energy_yielding	0.00	0.36
nitrite_denitrification	0.00	0.36
human_pathogens_septicemia	0.00	0.36
human_pathogens_pneumonia	0.00	0.36
anoxygenic_photoautotrophy_Fe_oxidizing	0.00	0.36
reductive_acetogenesis	0.00	0.36
nitrite_ammonification	0.66	0.24
anammox	0.00	0.24
denitrification	0.00	0.24
nonphotosynthetic_cyanobacteria	0.00	0.24
human_pathogens_gastroenteritis	0.66	0.12
acetoclastic_methanogenesis	0.00	0.12
methanogenesis_by_disproportionation_of_methyl_groups	0.00	0.12
methanogenesis_using_formate	0.00	0.12
methanogenesis_by_CO2_reduction_with_H2	0.00	0.12
methanogenesis_by_reduction_of_methyl_compounds_with_H2	0.00	0.12
methanogenesis	0.00	0.12
dissimilatory_arsenate_reduction	0.00	0.12
arsenite_oxidation_detoxification	0.00	0.12
human_pathogens_nosocomia	0.00	0.12
human_pathogens_meningitis	0.00	0.12
human_pathogens_diarrhea	0.00	0.12
fish_parasites	0.00	0.12
nitrogen_respiration	0.00	0.12
chlorate_reducers	0.00	0.12
photosynthetic_cyanobacteria	0.00	0.12
anoxygenic_photoautotrophy	0.00	0.12
hydrogenotrophic_methanogenesis	0.00	0.00
nitrification	0.00	0.00
respiration_of_sulfur_compounds	0.00	0.00
dissimilatory_arsenite_oxidation	0.00	0.00
human_associated	0.00	0.00
mammal_gut	0.00	0.00
chloroplasts	0.00	0.00
oxygenic_photoautotrophy	0.00	0.00
photoautotrophy	0.00	0.00
phototrophy	0.00	0.00
